# Future research and therapeutic applications of human stem cells: general, regulatory, and bioethical aspects

**DOI:** 10.1186/1479-5876-8-131

**Published:** 2010-12-10

**Authors:** Antonio Liras

**Affiliations:** 1 Department of Physiology, School of Biological Sciences, Complutense University of Madrid, Spain

## Abstract

There is much to be investigated about the specific characteristics of stem cells and about the efficacy and safety of the new drugs based on this type of cells, both embryonic as adult stem cells, for several therapeutic indications (cardiovascular and ischemic diseases, diabetes, hematopoietic diseases, liver diseases). Along with recent progress in transference of nuclei from human somatic cells, as well as iPSC technology, has allowed availability of lineages of all three germ layers genetically identical to those of the donor patient, which permits safe transplantation of organ-tissue-specific adult stem cells with no immune rejection. The main objective is the need for expansion of stem cell characteristics to maximize stem cell efficacy (i.e. the proper selection of a stem cell) and the efficacy (maximum effect) and safety of stem cell derived drugs. Other considerations to take into account in cell therapy will be the suitability of infrastructure and technical staff, biomaterials, production costs, biobanks, biosecurity, and the biotechnological industry. The general objectives in the area of stem cell research in the next few years, are related to identification of therapeutic targets and potential therapeutic tests, studies of cell differentiation and physiological mechanisms, culture conditions of pluripotent stem cells and efficacy and safety tests for stem cell-based drugs or procedures to be performed in both animal and human models in the corresponding clinical trials. A regulatory framework will be required to ensure patient accessibility to products and governmental assistance for their regulation and control. Bioethical aspects will be required related to the scientific and therapeutic relevance and cost of cryopreservation over time, but specially with respect to embryos which may ultimately be used for scientific uses of research as source of embryonic stem cells, in which case the bioethical conflict may be further aggravated.

## Introduction

A great interest has arisen in research in the field of stem cells, which may have important applications in tissue engineering, regenerative medicine, cell therapy, and gene therapy because of their great therapeutic potential, which may have important applications [1,2].

Cell therapy is based on transplantation of live cells into an organism in order to repair a tissue or restore lost or defective functions. Cells mainly used for such advanced therapies are stem cells, because of their ability to differentiate into the specific cells required for repairing damaged or defective tissues or cells [3]. Regenerative medicine is in turn a multidisciplinary area aimed at maintenance, improvement, or restoration of cell, tissue, or organ function using methods mainly related to cell therapy, gene therapy, and tissue engineering.

There is however much to be investigated about the specific characteristics of stem cells. The mechanisms by which they differentiate and repair must be understood, and more reliable efficacy and safety tests are required for the new drugs based on stem cells.

### General aspects of stem cells

The main properties that characterize stem cells include their indefinite capacity to renew themselves and leave their initial undifferentiated state to become cells of several lineages. This is possible because they divide symmetrically and/or asymmetrically, i.e. each stem cell results in two daughter cells, one of which preserves its potential for differentiation and self-renewal, while the other cell directs itself toward a given cell lineage, or they both retain their initial characteristics.

Stem cells are able to renew themselves and produce mature cells with specific characteristics and functions by differentiating in response to certain physiological stimuli. Different types of stem cells are distinguished based on their potential and source. These include the so-called *totipotent *embryonic cells, which appear in the early stages of embryo development, before blastocyst formation, capable of forming a complete organism, as well as all intra and extra embryonic tissues. There are also *pluripotent *embryonic cells, which are able to differentiate into any type of cell, but not into the cells forming embryonic structures such as placenta and umbilical cord. *Multipotent *adult cells (such as hematopoietic cells, which may differentiate into platelets, red blood cells, or white blood cells) are partially specialized cells but are able to form a specific number of cell types. *Unipotent *cells only differentiate into a single cell lineage, are found in the different body tissues, and their function is to act as cell reservoirs in the different tissues. Germ stem cells are pluripotent embryonic stem cells derived from gonadal buds of the embryo which, after a normal embryonic development, will give rise to oocytes and spermatozoa [4,5].

In the fetal stage there are also stem cells with differentiation and self-renewal abilities. These stem cells occur in fetal tissues and organs such as blood, liver, and lung and have similar characteristics to their counterparts in adult tissues, although they show a greater capacity to expand and differentiate [6]. Their origin could be in embryonic cells or in progenitors unrelated to embryonic stem cells.

Adult stem cells are undifferentiated cells occurring in tissues and organs of adult individuals which are able to convert into differentiated cells of the tissue where they are. They thus act as natural reservoirs for replacement cells which are available throughout life when any tissue damage occurs. These cells occur in most tissues, including bone marrow, trabecular bone, periosteum, synovium, muscle, adipose tissue, breast gland, gastrointestinal tract, central nervous system, lung, peripheral blood, dermis, hair follicle, corneal limbus, etc. [7].

In most cases, stem cells from adult tissues are able to differentiate into cell lineages characteristics of the niche where they are located, such as stem cells of the central nervous system, which generate neurons, oligodendrocytes, and astrocytes. Some unipotent stem cells, such as those in the basal layer of interfollicular epidermis (producing keratinocytes) or some adult hepatocytes, may even have a repopulating function in the long term [8].

Adult stem cells have some advantages in terms of clinical applications over embryonic and induced pluripotent stem cells because their use poses no ethical conflicts nor involves immune rejection problems in the event of autologous implantation, but induced pluripotent stem cells are at least, if not more capable than those from adult (stem) cells.

#### Mesenchymal stem cells

Although adult stem cells are primarily unipotent cells, under certain conditions they show a plasticity that causes them to differentiate into other cell types within the same tissue. Such capacity results from the so-called transdifferentiation in the presence of adequate factors--as occurs in mesenchymal stem cells, which are able to differentiate into cells of an ectodermal (neurons and skin) and endodermal (hepatocytes, lung and intestinal cells) origin--or from the cell fusion process, such as *in vitro *fusion of mesenchymal stem cells with neural progenitors or *in vivo *fusion with hepatocytes in the liver, Purkinje neurons in the brain, and cardiac muscle cells in the heart [9].

This is why one of the cell types most widely used to date in cell therapy are mesenchymal stem cells (MSCs), which are of a mesodermal origin and have been isolated from bone marrow, umbilical cord blood, muscle, bone, cartilage, and adipose tissue [10]. From the experimental viewpoint, the differential characteristics of MSCs include their ability to adhere to plastic when they are cultured *in vitro*; the presence of surface markers typical of mesenchymal cells (proteins such as CD105, CD73, and CD90) and the absence of markers characteristic of hematopoietic cells, monocytes, macrophages, or B cells; and their capacity to differentiate *in vitro *under adequate conditions into at least osteoblasts, adipocytes, and chondroblasts [11,12].

Recent studies have shown that MSCs support hematopoiesis and immune response regulation [13]. They also represent an optimum tool in cell therapy because of their easy *in vitro *isolation and expansion and their high capacity to accumulate in sites of tissue damage, inflammation, and neoplasia. MSCs are therefore useful in regenerative therapy, in graft-versus-host disease and in Crohn's disease, or in cancer therapy [14-17].

The development in the future of an optimum methodology for genetic manipulation of MSCs may even increase their relevant role in cell and gene therapy [18].

#### Adipose-derived mesenchymal stem cells

Bone marrow has been for years the main source of MSCs, but bone marrow harvesting procedure is uncomfortable for the patient, the amount of marrow collected is scarce, and the proportion of MSCs it contains as compared to the total population of nucleated cells is very low (0.001%-0.01%) [19]. By contrast, subcutaneous adipose tissue is usually abundant in the body and is a waste product from the therapeutic and cosmetic liposuctions increasingly performed in Western countries. These are simple, safe, and well tolerated procedures, with a complication rate of approximately 0.1%, where an amount of fat ranging from a few hundreds of milliliters to several liters (up to 3 liters, according to the recommendation of the World Health Organization) is usually drawn and subsequently discarded. Despite the suction forces exerted during aspiration, it is estimated that 98%-100% of tissue cells are viable after extraction. The liposuction method is therefore the most widely accepted for MSCs collection [20,21].

Adipose-derived stem cells (ASCs) were first identified in 2001 by Zuk et al. [22]. In addition to having the differentiation potential and self-renewal ability characteristic of MSCs, these cells secrete many cytokines and growth factors with anti-inflammatory, antiapoptotic, and immunomodulatory properties such as vascular endothelial growth factor (VEGF), hepatocyte growth factor (HGF), and insulin-like growth factor-1 (IGF-1), involved in angiogenesis, healing, and tissue repair processes [23]. This ability to secrete proangiogenic cytokines makes ASCs optimum candidates for cell therapy of ischemic diseases. In this regard, in a lower limb ischemia model in rats, intravenous or intramuscular ASCs administration was reported to significantly improve blood flow, probably due to the direct effect of ASCs differentiation into endothelial cells, and to the indirect effect of secretion of growth factors that promote neovascularization [24,25].

The immunomodulatory properties of ASCs and their lack of expression of MHC class II antigens also make them adequate for allogeneic transplantation, minimizing the risk of rejection. ASCs regulate T cell function by promoting induction of suppressor T cells and inhibiting production of cytotoxic T cells, NK cells, and proinflammatory cytokines such as tumor necrosis factor-α (TNF-α), interferon-γ (IFN-γ), and interleukine-12 (IL-12). These effects, complemented by secretion of soluble factors such as IL-10, transforming growth factor-β (TGF-β) and prostaglandin E2, account for the immunosuppressive capacity of these cells, which was demonstrated in a clinical trial where graft-versus-host disease (GVDH) was treated by intravenous injection of ASCs [26-28]. This immunosuppressive role of ASCs and their adjuvant effect in healing are also reflected in the encouraging results which are being achieved in various clinical trials investigating ASCs transplantation for treating fistula in patients with Crohn's disease [29] and radiotherapy-induced chronic ulcers [30].

Many other studies being conducted in animal models show the potential of ASCs to regenerate cranial bone, periodontal tissue and joint cartilage, in functional repair of myocardial infarction, and in stroke [31,32].

#### Other types of stem cells

*Hematopoietic stem cells *together with mesenchymal stem cells, the so-called "side population", and multipotent adult progenitor cells (MAPCs), are the stem cells forming bone marrow [33]. Their role is maintenance and turnover of blood cells and immune system.

The high rate of regeneration of the liver, as compared to other tissues such as brain tissue, is due to proliferation of two types of liver cells, hepatocytes, and *oval cells *(stem cells). In response to acute liver injuries (hepatectomy or hepatotoxin exposure), hepatocytes regenerate damaged tissue, while oval cells are activated in pathological conditions where hepatocytes are not able to divide (acute alcohol poisoning, phenobarbital exposure, etc.), proliferating and converting into functional hepatocytes [34].

In skeletal muscle, the stem cells, called *satellite cells*, are in a latent state and are activated following muscle injury to proliferate and differentiate into muscle tissue. *Muscle-derived stem cells *have a greater ability for muscle regeneration [35]. In cardiac tissue, *cardiac progenitor cells *are multipotent and may differentiate both *in vitro *and *in vivo *into cardiomyocytes, smooth muscle cells, and vascular endothelial cells [36,37].

*Neuronal stem cells *able to replace damaged neurons have been reported in the nervous system of birds, reptiles, mammalians, and humans. They are located in the dentate fascia of hippocampus and the subventricular area of lateral ventricles [38,39]. Stem cells have also recently been found in the peripheral nerve system (in the carotid body) [40]. Astrocytes, which are glial cells, have been proposed as multipotent stem cells in human brain [41].

The high renewal capacity of the skin is due to the presence in the epidermis of stem cells acting as a cell reservoir. These include *epidermal stem cells*, mainly located in the protuberance of hair follicle and which are capable of self-renewal for long time periods and differentiation into specialized cells, and *transient amplifying cells*, distributed throughout basal lamina and showing *in vivo *a very high division rate, but having a lower differentiation capacity [42].

#### Induced pluripotent stem cells

Induced pluripotent stem cells (iPSCs) from somatic cells are revolutionizing the field of stem cells. Obtained by reprogramming somatic stem cells of a patient through the introduction of certain transcription factors [43-48], they have a potential value for discovery of new drugs and establishment of cell therapy protocols because they show pluripotentiality to differentiate into cells of all three germ layers (endoderm, mesoderm, and ectoderm).

The iPSC technology offers the possibility of developing patient-specific cell therapy protocols [49] because use of genetically identical cells may prevent immune rejection. In addition, unlike embryonic stem cells, iPSCs do not raise a bioethical debate, and are therefore a "consensus" alternative that does not require use of human oocytes or embryos [50]. Moreover, iPSCs are not subject to special regulations [51] and have shown a high molecular and functional similarity to embryonic cells [52,53].

Highly encouraging results have been achieved with iPSCs from skin fibroblasts, differentiated to insulin-secreting pancreatic islets [54]; in lateral amyotrophic sclerosis (Lou Gehrig disease) [55]; and in many other conditions such as adenosine deaminase deficiency combined with severe immunodeficiency, Shwachman-Bodian-Diamond syndrome, type III Gaucher disease, Duchenne and Becker muscular dystrophy, Parkinson and Huntington disease, diabetes mellitus, or Down syndrome [56]. Good results have also been reported in spinal muscular atrophy [57] and in screening tests in toxicology and pharmacology, for toxic substances for the embryo or for teratogenic substances [58].

A very recent application has been reported by Moretti et al. [59] in the long QT syndrome, a hereditary disease associated to prolongation of the QT interval and risk of ventricular arrhythmia. iPCSs retain the genotype of type 1 disease and generate functional myocytes lacking the *KCNQ1 *gene mutation. Patients show normalization of the ventricular, atrial, and nodal phenotype, and positively express various normal cell markers.

### Stem cell therapy: A new concept of medical application in Pharmacology

For practical purposes, human embryonic stem cells are used in 13% of cell therapy procedures, while fetal stem cells are used in 2%, umbilical cord stem cells in 10%, and adult stem cells in 75% of treatments. To date, the most relevant therapeutic indications of cell therapy have been cardiovascular and ischemic diseases, diabetes, hematopoietic diseases, liver diseases and, more recently, orthopedics [60]. For example, more than 25,000 hematopoietic stem cell transplantations (HSCTs) are performed every year for the treatment of lymphoma, leukemia, immunodeficiency illnesses, congenital metabolic defects, hemoglobinopathies, and myelodysplastic and myeloproliferative syndromes [61].

Depending on the characteristics of the different therapeutic protocols and on the requirements of each condition, each type of stem cell has its advantages and disadvantages. Thus, embryonic stem cells have the advantages of being pluripotent, easy to isolate, and highly productive in culture, in addition to showing a high capacity to integrate into fetal tissue during development. By contrast, their disadvantages include immune rejection, the possibility that they differentiate into inadequate cell types or induce tumors, and contamination risks. Germ stem cells are also pluripotent, but the source from which they are harvested is scarce, and they may develop embryonic teratoma cells *in vivo*. Adult stem cells are multipotent, have a greater differentiation potential, less likely to induce no immune rejection reactions, and may be stimulated by drugs. Their disadvantages include that they are scarce and difficult to isolate, grow slowly, differentiate poorly in culture, and are difficult to handle and produce in adequate amounts for transplantation. In addition, they behave differently depending on the source tissue, show telomere shortening, and may carry the genetic abnormalities inherited or acquired by the donor.

These disadvantages of adult stem cells are less marked in some of the above mentioned subtypes, such as mesenchymal stem cells obtained from bone marrow or adipose tissue, or iPSCs. In these cases, harvesting and production are characterized by their easiness and increased yield rates in the growth of the cultures. Their growth is slow but meets experimental requirements, and their differentiation and implantation are highly adequate [62,63].

Overall, at least three types of therapeutic strategies are considered when using stem cells. The first is stimulation of endogenous stem cells using growth factors, cytokines, and second messengers, which are able to induce self-repair of damaged tissues or organs. The second alternative is direct administration of stem cells so that they differentiate at the damaged or non-functional tissue sites. The third possibility is transplantation of cells, tissues, or organs taken from cultures of stem cell-derived differentiated cells.

The *US Food and Drug Administration *defines somatic cell therapy as the administration of autologous, allogeneic, or xenogeneic non-germ cells--excluding blood products for transfusion--which have been manipulated or processed and propagated, expanded, selected *ex vivo*, or drug-treated.

Cell therapy applications are related to the treatment of organ-specific diseases such as diabetes or liver diseases. Cell therapy for diabetes is based on islet transplantation into the portal vein of the liver and results in an improved glucose homeostasis, but graft function is gradually lost in a few years after transplantation. Liver diseases (congenital, acute, or chronic) may be treated by hepatocyte transplantation, a technique under development and with significant disadvantages derived from difficulties in hepatocyte culture and maintenance. The future here lies in implantation of hepatic stem cells, or in implantation of hepatic cells obtained by differentiation of a different type of stem cell, such as mesenchymal stem cells.

Other applications, still in their first steps, include treatment of hereditary monogenic diseases such as hemophilia using hepatic sinusoidal endothelial cells [64] or murine iPSCs obtained by fibroblast differentiation into endothelial cells or their precursors [65]. As regards hemophilia, an optimum candidate because it is a monogenic disease and requires low to moderate expression levels of the deficient coagulation factor to achieve a moderate phenotype of disease, great progress is being made in both gene therapy and cell therapy using viral and non-viral vectors. The Liras et al. group has reported encouraging preliminary results using non-viral vectors and mesenchymal stem cells derived from adult human adipose tissue [66-68].

Very recently, Fagoonee et al. [69] first showed that adult germ line cell-derived pluripotent stem cells (GPSCs) may differentiate into hepatocytes *in vitro*, which offers a great potential in cell therapy for a very wide variety of liver diseases.

#### Histocompatible stem cell therapy

Since one of the most important applications of cell therapy is replacement of the structure and function of damaged or diseased tissues and organs, avoidance or reduction of rejection due to a natural immune response of the host to the transplant is a highly relevant consideration. Recent progress in nuclear transference from human somatic cells, as well as the iPSC technology, have allowed for availability of lineages of all three germ layers genetically identical to those of the donor patient, which permits safe transplantation of organ-tissue-specific adult stem cells with no immune rejection [70].

On the other hand, adipose-derived mesenchymal stem cells (ASCs) are able to produce adipokines, including many interleukines [71]. ASCs also have immunosuppressive capacity, regulating inflammatory processes and T-cell immune response [72-74]. The lack of HLA-DR expression and immunosuppressive properties of ASCs make these cells highly valuable in allogeneic transplantation to prevent tissue rejection. They do not induce alloreactivity *in vitro *with incompatible lymphocytes and suppress the antigen response reaction by lymphocytes. These findings support the idea that ASCs share immunosuppressive properties with bone marrow-derived MSCs and may therefore represent a new alternative for conditions related to the immune system [75-77].

### Suitability of infrastructure and technical staff

Any procedure related to cell therapy requires a strict control of manipulation and facilities. In addition, it should not be forgotten that cell therapy products are considered as drugs, and the same or a similar type of regulation should therefore be followed for them.

Products must be carefully detailed and described, stating whether autologous, allogeneic, or xenogeneic cells are administered. Xenogeneic cells are included by the *US Food and Drug Administration *[78] as human cells provided there has been *ex vivo *contact with living non-human cells, tissues, or organs. It should also be detailed whether cells have been manipulated together with other non-cell materials such as synthetic or natural biomaterials, with other types of materials or agents such as growth factors or serum.

As regards the production process, a detailed description must be given of all procedures related to product quality in the *Standard Operating Procedures *(SOPs), as for conventional medical products. The purity, safety, functionality, and identity criteria used for conventional drugs must be met.

Because of the characteristics of these products, their storage period before sale or distribution should necessarily be shorter, as they cannot obviously be subject to prior sterilization (hence the use of cryopreservation as the most adequate storage method). Therefore, the production process must occur in a highly aseptic environment with comprehensive controls of both raw materials and handlers. Needless to say that production process should be highly reproducible and validated both on a small scale for a single patient and on a large scale. For an autologous therapy procedure, cell harvesting from the patient will be aimed at collecting healthy cells whenever this is possible, because in some cases, if no mosaicism exists and the disease is inherited, all cells will carry the relevant mutation, in which case this procedure will not be feasible. In hemophilia the situation may be favorable, because mosaicism is found in 30% of the cases [79].

Cell therapy products should adhere to the Current *Good Manufacturing Practices*, including *quality control *and *quality assurance *programs, which establish minimum quality requirements for management, staff, equipment, documentation, production, quality control, contracting-out, claims, product recall, and self-inspection. Production and distribution should be controlled by the relevant local or national authorities based on the *International Conference on Harmonization of Pharmaceuticals for Human Use*, which standardizes the potential interpretations and applications of the corresponding recommendations [80].

It is of paramount importance to prevent potential contamination, both microbiological and by endotoxins, due to defects in environmental conditions, handlers, culture containers, or raw materials, or crossed contamination with other products prepared at the same production plant. Care should be taken with methods for container sterilization and control of raw materials and auxiliary reagents, use of antibiotics, use of High Efficiency Particulate Absorbing (HEPA) filters to prevent airborne cross-contamination, separate handling of materials from different patients, etc.

In compliance with official standard books such as the *European Pharmacopoeia *(Eur.Ph.) [81] or *the United States Pharmacopeia *(USP) [82], each batch of a biological product should pass a very strict and specific test depending on the characteristics of the cell therapy product, such as colorimetry, oxygen consumption, or PCR. Facilities where products are handled, packaged, and stored should be designed and organized according to the guideline *Good Manufacturing Practice for Pharmaceutical Manufacturers *(GMP) [83]. The most important rooms of these facilities include the so-called *clean rooms*, which are classified in four classes (A-D) depending on air purity, based on the number of particles of two sizes (≥ 0.5 μm, ≥ 5 μm). Other parameters such as temperature, humidity, and pressure should be taken into account and monitored because of their potential impact on particle generation and microorganism proliferation.

As regards to the number of technical staff, this should be the minimum required and should be especially trained in basic hygiene measures required for manipulation in a clean room. Material and staff flows should be separated and be unidirectional to minimize cross contamination, and control and documentation of all activities is necessary. Technical staff should have adequate qualification both for the conduct and surveillance of all activities.

*Good Manufacturing Practice for Pharmaceutical Manufacturer*s is a general legal requirement for all biological medicinal products before their marketing or distribution. As in tissue donation, use of somatic cells from a donor requires the method to be as least invasive as possible and to be always performed after obtaining signed informed consent. In this regard, risk-benefit assessment in this field is even more necessary in this field than in other areas because of the sometimes high underlying uncertainty when stem cells are used [84].

### Biomaterials for Cellular Therapy

In advanced therapies, particularly in cell therapy and tissue engineering, the biomaterial supporting the biological product has a similar or even more important role as the product itself. Such biomaterials serve as the matrix for nesting of implanted cells and tissues because they mimic the functions of the tissue extracellular matrix.

Biomaterials for cell therapy should be biocompatible to prevent immune rejection or necrosis. They should also be biodegradable and assimilable without causing an inflammatory response, and should have certain structural and mechanical properties. Their primary role is to facilitate location and distribution of somatic cells into specific body sites--in much the same way as excipients in classical pharmacology--and to maintain the three-dimensional architecture that allows for formation and differentiation of new tissue.

Materials may be metals, ceramic materials, natural materials, and synthetic polymers, or combinations thereof. Synthetic polymers are biocompatible materials (although less so than natural materials) whose three-dimensional structure may easily and reproducibly be manufactured and shaped. Their degradation rate may be controlled, they are free from pathogens, and bioactive molecules may be incorporated into them. Their disadvantage is that they may induce fibrous encapsulation. Natural polymers such as collagen, alginate, or keratin extracts are also biocompatible and, as synthetic polymers, may be incorporated active biomolecules. They have however the disadvantages that they may mimic the natural structure and composition of extracellular matrix, their degradation rate is not so easy to control, have less structural stability, are sensitive to temperature, and may be contaminated by pathogens.

In any case, use of one or the other type of biomaterial is always related to the administration route in cell therapy protocols, *implantation *or *injection*. Thus, in the *injection*-based procedure, which is simpler and requires no surgery but can only be used for certain areas, biomaterials are usually in a hydrogel state, forming a hydrophilic polymer network, as occurs in PEO (*polyethylene oxide*), PVA (*polyvinyl alcohol*), PAA (*polyacrylic acid*), agarose, alginate, collagen, and hyaluronic acid.

Research on biomaterials for cell therapy is aimed not only at finding or synthesizing new materials, but also at designing methods that increase their efficacy [85]. For example, control of the porous structure of these materials is very important for increasing their efficacy in tissue regeneration (through *solvent casting/particulate leaching, freeze-drying, fiber bonding, electrospinning, melt molding, membrane lamination, or hydrocarbon templating*). An attempt may also be made to increase biocompatibility through chemical (oxidation or hydrolysis) or physical modification. To increase cell adhesion and protein adsorption, water-soluble polymers may be added to the biomaterial surface. Bioactive molecules such as enzymes, proteins, peptides, or antibodies may also be coupled, as is the standard and routine practice, to the biomaterial surface to provide it with functionality. Other substances such as cytokines or growth factors which promote migration, proliferation, or overall function of cells used in therapy may be coupled. Another highly relevant line of research aims at minimizing immune rejection when cells to be used are not autologous cells. *Immunoisolation *by cell microencapsulation (coating of biologically active products by a polymer matrix surrounded by a semipermeable membrane), which allows for two-directional substance diffusion, is extremely important and is giving optimal results [86-89].

Many types of biomaterials are being developed for bone tissue regeneration based on either demineralized bone matrix or in bladder submucosa matrix combined with poly(lactic-co-glycolic acid) (PLGA), which accelerates regeneration and promotes cell accommodation in *in vivo *bone formation [90,91]. For bypass procedures in large-diameter vessels, synthetic polymers such as expanded *polytetrafluoroethylene *(ePTFE) or *polyethylene terephthalate *(PET) fiber have been applied [92]. For peripheral nerve repair, use of axonal guides made of several materials such as silicone, collagen, and PLGA [93], and recently of Schwann cells to accelerate axonal regeneration, have been reported [94].

Advances in identification of the optimal characteristics of the matrix and an increased understanding of interactions between cells and biomaterials will condition development of future cell therapy protocols.

### Production costs, biobanks and biosecurity in cell therapy

Production costs in cell therapy are high (currently, a treatment may cost more than 40,000 dollars), mainly because drug products based on cell therapy are prepared on a low and almost individual scale, but allogeneic procedures [95] and availability of cryopreserved cell banks (biobanks) will lead cell therapy to occupy a place in the market of future pharmacology.

Costs are accounted for by different items, all of them necessary, including multiple surgical procedures, maintenance of strict aseptic conditions, specific training of technical staff and maintenance of overall technical and staff support, specialized facilities, the need for producing small and highly unstable batches and, of course, design and development of the different market strategies. The question arises as to whether these costs will be compatible with at least partial funding by governments, medical insurance companies, and public and private health institutions, and with current and future demographic movements ("demographic" patients) [96].

Until widespread use of allogeneic protocols becomes established, thus overcoming the problems derived from immune rejection, and although it is not certain if allogeneic cell transplantation will ever be free from clinical complications, biobanks represent the hope for the project of cell therapy to become a reality in the future [97]. Concerning production costs, even if biobanks exist, the production of cellular therapies often require the use of cytokines, growth factors and specialized reagents which are very expensive.

*Stem cell banks *[98] store lines of embryonic and adult human stem cells for purposes related to biomedical research. Regardless of their public (nonprofit, anonymous donation) or private (donation limited to a client's environment) nature, stem cell banks may store cell lines from umbilical cord and placental tissue, rich in hematopoietic stem cells, or cell lines derived from various somatic tissues, either differentiated or not. There are banks of cryopreserved umbilical cord bloods throughout Europe and North America. These were set up primarily for hematopoietic stem cell transplantation, but they are available for other clinical uses.

Two of the most relevant international banks are the *US National Stem Cell Bank *(NSCB) [99] and the *United Kingdom Stem Cell Bank *[100].

The NSCB was set up at the WiCell Research Institute on September 2005 and is devoted to acquisition, characterization, and distribution of 21 embryonic stem cell lines and their subclones for use in research programs funded by the National Institute of Health (NIH), and to provide the research community with adequate technical support. The UKSCB was created on September 2002 as an independent initiative of the Medical Research Council (MRC) and the Biological Sciences Research Council (BBSRC), and serves as a storage facility for cell lines from both adult and embryonic stem cells which are available for use in basic research and in development of therapeutic applications.

Culture of adult stem cells, which are safer to use, must be kept in culture since they are harvested until they are used. This may involve risks of contamination or pseudodifferentition leading to a loss of biological specificity of each target cell population in its physiological interaction with all other tissues. This makes it essential, for biosafety purposes, to assess and monitor any *ex vivo *differentiation procedure, first *in vitro *cultures and then in animal models, to verify the properties of the stem cell and its genetic material and to prevent risks, which may range from tumor formation to simple uncertainty about its differentiation [101].

If the biological material is human embryonic stem cells (hES), there is no standard method for characterization, but some of their specific characteristics may be assessed, including the nucleus-cytoplasm ratio, number of nucleoli and morphological characteristics of the colony, growth rate, percent clonogenicity, *in vitro *embryoid body formation, and *in vitro *teratoma formation after subcutaneous implantation in immunodeficient mice. Clinical use of this type of cells always requires control of their *in vitro *differentiation into multipotent or fully differentiated cells with tumorogenic potential. The cell characterization process in molecular and cellular terms is time-consuming and takes some years, especially as regards self-renewal pathways and development potential, which are very different in humans and murine models.

Control of cell transformation is particularly important for biosecurity of cell therapy products. Hematopoietic stem cells are extremely resistant to transformation due to two types of control, replicative senescence (phase M1) and cell crisis (phase M2). Cell senescence is usually induced by a moderate telomere shortening or by oncogene expression leading to morphological changes such as cell lengthening or a change in expression of specific senescence markers. The cell crisis phase occurs when some cell types avoid this control until telomeres become critically short, chromosomes become unstable, and apoptosis is activated. Spontaneous transformations have been reported in human (hMSCs) and murine (mMSCs) mesenchymal stem cells [102], suggesting that extreme caution is required when these cells are used in clinical treatments. However, it should also be noted that cell transformation occurs after a long time period (4 months), much longer than the culture periods of therapeutic cells (2-14 passages; 1-8 weeks), which is the minimum and almost sufficient time to obtain an adequate number of cells for a cell therapy treatment, and during which the senescence phenomenon is less likely.

#### Biotechnological industry

Stem cell research is in its early stages of development, and the market related to cell therapy is therefore highly immature, but the results achieved to date raise great expectations.

In order to analyze the current status and perspectives of this particular market, a distinction should be made between embryonic and adult stem cells, because the number of companies in these two fields is very different (approximately 30-40 working with adult versus 8-10 working with embryonic stem cells). Such difference is mainly due to ethical and legal issues associated to each cell type or to the disparity of criteria between the different countries regarding the industrial and even intellectual properties of the different technologies derived from stem cell research.

Overall, the potential numbers of patients who could benefit from cell therapy in the US would be approximately 70 million patients with cardiovascular disease, 50 millions with autoimmune diseases, 18 millions with diabetes, 10 millions with cancer, 4.5 millions with Alzheimer's disease, 1 million with Parkinson's disease, 1.1 millions with burns and wounds, and 0.15 millions with medullary lesions (data taken from Advanced Cell Technology [103].

Today, many pharmaceutical companies, including the big ones, are reluctant to enter this market because of the great investment required and because a very hard competition is expected in the pharmaceutical market. To date, the most profitable strategy has been the signing of agreements between big pharmaceutical companies and other small biotechnological companies whose activity is 100% devoted to cell therapy and regenerative medicine.

Special mention should be made of induced pluripotent stem cells (iPSCs), which have raised great expectations in the pharmaceutical industry because products to be derived from them, as noted above, will be applicable in a very wide range of development of new drugs and new procedures for the treatment of a great number of human diseases. At least 5-10 years will elapse until these products, not therapeutic yet and under study, are in therapeutic use and yield an economic return to biotechnological companies. Today, this interesting potential of therapeutic products derived from iPSCs still faces great technical and scientific challenges, and a very long time will be required until they fulfill their promise.

Overall, business models for marketing must be well devised and optimized, and also very well tested and based on accumulated experience with the various types of both adult and embryonic or induced stem cells [104].

### Research perspectives of stem cells

The general objectives in the area of stem cell research in the next few years, are related to identification of therapeutic targets and potential therapeutic tests. Within these general objectives, other specific objectives will be related to studies of cell differentiation and cellular physiological mechanisms that will enhance understanding, prevention, and treatment of some congenital or acquired defects. Other objectives would be to establish the culture conditions of pluripotent stem cells using reliable cytotoxicity tests and the optimum type of cell or tissue to be transplanted depending on the disease to be treated (bone marrow for leukemia and chemotherapy; nerve cells for treating conditions such as Parkinson and Alzheimer diseases; cardiac muscle cells for heart diseases, or pancreatic islets for the treatment of diabetes.

The current reality is that, although extensive research is ongoing and encouraging partial results are being achieved, there is still much to be known about the mechanisms of human development and all differentiation processes involved in the whole process from fertilization to the full development of an organism. In this, which appears so simple, lies the "mystery" surrounding differentiation of the different stem cells and the many factors that condition it.

A second pending question, is the efficacy and safety tests for stem cell-based drugs or procedures to be performed in both animal and human models in the corresponding phase I-III clinical trials.

The final objective of stem cell research is to "cure" diseases. Theoretically, stem cell therapy is one of the ideal means to cure almost all human diseases known, as it would allow for replacing defective or dead cells by normal cells derived from normal or genetically modified human stem cell lines [105].

If, as expected, such practices are possible in the future, stem cell research will shift the paradigm of medical practice. Some scientists and healthcare professionals think, for example, that Parkinson's disease, spinal cord injury, and type 1 diabetes [106] may be the first candidates for stem cell therapy. In fact, the *US Food and Drug Administration *has already approved the first clinical trial of products derived from human embryonic stem cells in acute spinal cord injuries [107,108].

Human stem cells, mainly autologous bone marrow cells, autologous and allogeneic mesenchymal cells, and some allogeneic neural cells, are currently being assessed in various clinical studies. As regards transplantation of bone marrow and mesenchymal cells, many data showing its safety are already available, while the efficacy results reported are variable. The most convincing likely explanation for this is that many mechanisms of action of these cells are not known in detail, which makes results unpredictable. Despite this, there is considerable optimism based on the immune suppression induced by mesenchymal stem cells and on their anti-inflammatory properties, which may be beneficial for many conditions such as graft-versus-host disease, solid organ transplantation, and pulmonary fibrosis. Variable results have been reported after use of mesenchymal stem cells in heart diseases, stroke, and other neurodegenerative disorders, but no significant effects were seen in most cases. By contrast, encouraging results were found in the correction of multiple sclerosis, at least in the short term. Neural stem cells may be highly effective in inoperable glioma, and embryonic stem cells for regeneration of pancreatic beta cells in diabetes [109].

The change in policy regarding research with embryonic stem cells by the Obama administration, which heralds a change of environment leading to an increased cooperation in the study and evaluation of stem cell therapies, opens up new and better expectations in this field. The initiative by the California Institute for Regenerative Medicine [110] has resulted in worldwide collaboration for these new drugs based on stem cells [111]. Thus, active participation of governments, research academies and institutes, pharmaceutical and biotechnological companies, and private investment may shape a powerful consortium that accelerates progress in this field to benefit of health.

### Legal and regulatory issues of cell therapy

Cell therapy is one of the *advanced therapy products *(ATPs), together with gene therapy and tissue engineering. A regulatory framework is required for ATPs to ensure patient accessibility to products and governmental assistance for their regulation and control. Certainty, scientific reality and objectivity, and flexibility to keep pace with scientific and technological evolution are the characteristics defining an effective regulation.

Aspects to be regulated mainly include control of development, manufacturing, and quality using release and stability tests; non-clinical aspects such as the need for studies on biodistribution, cell viability and proliferation, differentiation levels and rates, and duration of *in vivo *function; and clinical aspects such as special dose characteristics, stratification risk, and specific pharmacovigilance and traceability issues.

#### European Medicines Agency: Regulation in the European Union

European countries may be classified into three groups based on their different positions regarding research with embryonic stem cells of human origin. i) Countries with a restrictive political model (Iceland, Lithuania, Denmark, Slovenia, Germany, Ireland, Austria, Italy, Norway, and Poland); ii) Countries with a liberal political model (Sweden, Belgium, United Kingdom, and Spain); and iii) Countries with an intermediate model (Latvia, Estonia, Finland, France, Greece, Hungary, Switzerland, the Netherlands, Bulgaria, Cyprus, Portugal, Turkey, Ukraine, Georgia, Moldavia, Romania, and Slovakia).

The *Seventh Framework Program for Research of the European Union*, coordinated by the European Medicines Agency, was approved on July 2006 [112]. This Seventh Framework Program provides for funding of research projects with embryonic stem cells in countries where this type of research is legally accepted, and the projects involving destruction of human embryos will not be financed with European funds. Guidelines on therapeutic products based on human cells are also established [113].

This regulation replaces the points in the prior 1998 regulation (CPMP/BWP/41450/98) referring to the manufacture and quality control of therapy with drugs based on human somatic cells, adapting them to the applicable law and to the heterogeneity of products, including combination products. Guidance is provided about the criteria and tests for all starting materials, manufacturing process design and validation, characterization of cell-base medicinal products, quality control aspects of the development program, traceability and vigilance, and comparison. Is also provides specific guidance of matrixes and stabilizing and structural devices or products as combination components.

The directive recognizes that conventional non-clinical pharmacology and toxicological studies may be different for cell-based drugs, but should be strictly necessary for predicting response in humans. It also establishes the guidelines for clinical trials as regards pharmacodynamic and pharmacokinetic studies, defining the clinically effective safe doses. The guideline describes the special consideration to be given to pharmacovigilance issues and the risk management plan for these products.

The guideline has therefore a multidisciplinary nature and addresses development, manufacture, and quality control, as well as preclinical and clinical development of medicinal products based on somatic cells (Directive 2001/83/EC) and tissue engineering products (Regulation 1394/2007/EC2). Includes autologous or allogeneic (but not xenogeneic) protocols based on cells either isolated or combined with non-cell components, or genetically modified. However, the document does not address non-viable cells or fragments from human cells.

Legislation on cell therapy in Europe is based on three Directives: Directive 2003/63/EC (amending Directive 2001/83/EC), which defines cell therapy products as clinical products and includes their specific requirements; Directive 2001/20/EC, which emphasizes that clinical trials are mandatory for such products and describes the special requirements for approval of such trials; and Directive 2004/23/EC, which establishes the standard quality, donation safety, harvesting, tests, processing, preservation, storage, and distribution of human tissues and cells.

The *marketing authorization *application has been prepared by the European Medicines Agency so that cell therapy products should meet the same administrative and scientific requirements as any other drug [114].

#### US Food and Drug Administration (FDA): Regulation in the United States of America

In the United States of America, restrictions are limited to research with federal funds. No limitations exist for research with human embryonic stem cells provided the funds come from private investors or specific states. In countries such as Australia, China, India, Israel, Japan, Singapore, and South Korea, therapeutic cloning is permitted.

The FDA has developed a regulatory framework that controls both cell- and tissue-based products, based on three general areas: i) Prevention of use of contaminated tissues or cells (e.g. AIDS or hepatitis); ii) prevention of inadequate handling or processing that may damage or contaminate those tissues or cells; and iii) clinical safety of all tissues or cells that may be processed, used for functions other than normal functions, combined with components other than tissues, or used for metabolic purposes. The FDA regulation, derived from the 1997 basic document "*Proposed approach to regulation of cellular and tissue-based products*" [115]. The FDA has recently issued updates to previous regulations referring to human cells, tissues, and all derived products [116]. This regulation provides an adequate regulatory structure for the wide range of stem cell-based products which may be developed to replace or repair damaged tissue, as both basic and clinical researchers and those working in biotechnological and pharmaceutical companies which need greater understanding and information to answer many questions before submitting a stem cell-based product for clinical use.

It should be reminded that, unlike conventional medicinal products, many stem cell-derived products are developed at universities and basic research institutions, where preclinical studies are also conducted, and that researchers there may not be familiar with the applicable regulations in this field. The FDA also provides specific recommendations on how scientists should address the safety and efficacy issues related to this type of therapies [117].

Any product based on stem cells or tissues undergoes significant processing, and it should therefore be fully verified that they retain their normal physiological function, either combined or not with other non-tissue components, because they will generally be used for metabolic purposes [116,118]. This is why many such products, if not all, must also comply with the Public Health Services Act, Section 351, governing the granting of licenses for biological products, which requires FDA submission and application for investigational protocols of new drugs before conducting clinical trials in humans.

The key points of the current FDA regulation for cell therapy products [117] include: i) demonstration of preclinical safety and efficacy; ii) no risk for donors of transmission of infectious or genetic diseases; iii) no risk for recipients of contamination or other adverse effects of cells or sample processing; iv) specific and detailed determination of the type of cells forming the product and what are their exact purity and potency; v) *in vivo *safety and efficacy of the product.

There is still much to be learned about the procedures to establish the safety and efficacy of cell therapy products. The greater the understanding of the biology of stem cell self-renewal and differentiation, the more precise the evaluation and prediction of potential risks. Development of techniques for cell identification within a mixed cell culture population and for follow-up of transplanted cells will also be essential to ascertain the potential *in vivo *invasive processes and to ensure safety.

Since new stem cell-based therapies develop very fast, the regulatory framework must be adapted and evolve to keep pace with such progress, although it may be expected to change more slowly. Meanwhile, the current regulations must provide the framework for ensuring the safety and efficacy of the next generations of stem cell-based therapeutic products.

### Bioethical aspects of cell therapy

Ethics is not in itself a discipline within human knowledge, but a "dialogue" where each person, from his/her stance, gives his/her opinion and listens to the other person's opinion.

Most cell therapy protocols have not been controversial. The exception is therapy with human embryonic stem cells, which has raised moral and ethical issues [119,120]. Such considerations refer to donor consent and problems associated to oocyte collection and the issue of destruction of human embryos [121].

Guidelines--ranging from total prohibition to controlled permissiveness--defining what may be permitted in research with pluripotent stem cells have been issued in countries all over the world [122].

All such guidelines reflect the different views about when life starts during the human embryonic development, as well as regulation of measures to protect oocyte donors and to reduce the probability of human embryo destruction [123].

There is general international agreement in that the results of stem cell research should not be applied in humans without prior ethical scrutiny. For this purpose, 42 European countries have national ethics committees since 2006, and a President's Council on Bioethics with an advisory role in bioethical matters was created in the US in 2001. The European Commission currently has the *Group on Ethics in Science and New Technologies*, an advisory, independent, and plural multidisciplinary body [124], and in other countries, such as the United Kingdom, legislation on action and bioethics is clearly established since several years ago [125].

The *Ethics and Health Team *at World Health Organization [126] acts as a permanent secretariat for the Global Summit of National Bioethics Commissions and cooperates with the *European Conference of National Ethics Committees *(COMETH) [127]. On the other hand, the UNESCO created in 1992 the *International Bioethics Committee *[128].

In the United States, the National Institute of Health provides detailed and updated information on various aspects related to stem cells [129] in order to educate and update on the different viewpoints on bioethical issues as a function of progress in science and technology related to the field of cell therapy.

The *National Academy of Sciences *issued in 2005 its first set of ethical standards for stem cell research [130], which were updated in 2007, 2008, and 2010, to adapt the guidelines to rapid scientific and political advances, by the *Human Embryonic Stem Cell Research Advisory Committee *created in 2006 with the support of the Ellison Medical Foundation, The Greenwall Foundation, and Howard Hughes Medical Institute. These updates and amendments have updated the guidelines of the different national academies and take into account the new role of the National Institute of Health with regard to research with human embryonic stem cells.

The *Presidential Commission on Bioethics *for the Study of Bioethical Issues advises President Obama on any bioethical issues that may arise from advances in biomedicine and in related areas of science and technology [131]. This commission works to identify and promote policies and practices ensuring ethically responsible actions in scientific research, health care, and technological innovation.

The *Kennedy Institute of Ethics at Georgetown University Library and Information Services *[132] allows for searching books, newspapers, journal articles, and other materials on bioethical issues. On the other hand, the *International Society for Stem Cell Research *[133] and, among others, the *Bioethics Advisory Committee *(BAC) *Singapore *[134] have set up ethical, legal, and social regulations derived from research in biomedical sciences and act as an advisory public service on stem cells.

In conclusion, scientists are aware of the need for ethical evaluation of their research. This is discussed in the *Declaration on Science and the Use of Scientific Knowledge *of the 1999 World Conference on Science held in Budapest, entitled Science for the Twenty-First Century: a New Commitment attests to this awareness [135]. This declaration states that scientific research and use of scientific knowledge should respect human rights and the dignity of human beings, in accordance with the Universal Declaration of Human Rights and the Universal Declaration on the Human Genome and Human Rights. The special responsibility of scientists for preventing uses of science which are ethically incorrect or have a negative impact for society is also established. Commitments are established [136] to teach the next generations of scientists that ethics and responsibility are part of their daily training and work, and to warn about any potential dilemmas that may arise in the future with the inexorable progress of science.

There are two general basic issues related to bioethics that should be considered with care and separately: First, *scientific and therapeutic relevance*, and second, the *cost of cryopreservation over time*. In term of relevance, it should be considered that cells should be useful for the treatment of a specific disease, but the exact time of their use is not known, and they therefore have to be cryopreserved. From a bioethical viewpoint, this is more questionable when dealing with embryos whose cryopreservation should be authorized by the parents and which will be used either for a particular use or for donation. These embryos may ultimately be used for scientific uses of research with embryonic stem cells, in which case the bioethical conflict may be further aggravated. The second aspect is the cost of cryopreservation. In some cases, such as preservation of umbilical cord blood, private biobanks are mainly used today, which may lead to a significant discrimination of people who cannot afford payment for such banks as compared to those who can.

Although ethical issues are less questionable in the case of adult stem cells as compared to embryonic stem cells, the *Council of Europe's Steering Committee on Bioethics *[137] has prepared an additional protocol, in the Convention on Human Rights and Biomedicine [138], which represents a general ethical and legal framework for signatory countries. This document details the different conditions, such as the prerequisite of approval by an independent committee competent in the corresponding field of a research project with both adult and embryonic stem cells assessing the relevance of the research purpose and the multidisciplinary aspects from the bioethical viewpoint. Signature by the donor, the research or hospital center, and the principal investigator of the project of an informed consent that explains in detail the potential risks and benefits and informs on the rights and safeguards, is also established as an indispensable condition.

## Conclusions

In recent decades, a great interest has arisen in research in the field of stem cells, which may have important applications in tissue engineering, regenerative medicine, and cell- and gene therapy. There is however much to be investigated about the specific characteristics of efficacy and safety of the new drugs based on this type of cells.

Cell therapy is based on transplantation of live cells into an organism in order to repair a tissue or restore lost or defective functions. Recent studies have shown that mesenchymal stem cells (MSCs) support hematopoiesis and immune response regulation and they represent an optimum tool in cell therapy because of their easy *in vitro *isolation and expansion and their high capacity to accumulate in sites of tissue damage, inflammation, and neoplasia. On the other hand, adipose-derived stem cells (ASCs) secrete many cytokines and growth factors with anti-inflammatory, antiapoptotic, and immunomodulatory properties. This makes these stem cells optimum candidates for cell therapy. Induced pluripotent stem cells (iPSCs) from somatic cells are revolutionizing the field of stem cells. They have a potential value for discovery of new drugs and establishment of cell therapy protocols because they show pluripotentiality to differentiate into cells of all three germ layers. The iPSC technology offers the possibility of developing patient-specific cell therapy protocols because use of genetically identical cells may prevent immune rejection, and unlike embryonic stem cells, iPSCs do not raise a bioethical debate, and are therefore a "consensus" alternative that does not require use of human oocytes or embryos.

Cell therapy applications are related to the treatment of organ-specific diseases such as diabetes or liver diseases. Another relevant application of cell therapy is development of cancer vaccines based on dendritic cells or cytotoxic T cells, in order to induce natural immunity. Other applications, still in their first steps, include treatment of hereditary monogenic diseases such as hemophilia. Until widespread use of allogeneic protocols becomes established, thus overcoming the problems derived from immune rejection, biobanks represent the hope for the project of cell therapy to become a reality in the future; control of cell transformation is also particularly important for biosecurity of cell therapy products.

Stem cell research is in its early stages of development, and the market related to cell therapy is therefore highly immature, but the results achieved to date raise great expectations. Today, many pharmaceutical companies, including the big ones, are reluctant to enter this market because of the great investment required and because very hard competition is expected in the pharmaceutical market. The general objectives in this area in the next few years, are related to identification of therapeutic targets and potential therapeutic tests. Within these general objectives, other specific objectives will be related to studies of cell differentiation and cellular physiological mechanisms that will enhance understanding, prevention, and treatment of some congenital or acquired defects. Other objectives would be to establish the culture conditions of pluripotent stem cells using reliable cytotoxicity tests and the optimum type of cell or tissue to be transplanted depending on the disease to be treated.

Up to now, most cell therapy protocols have not been controversial. The exception is therapy with human embryonic stem cells, which has raised moral and ethical issues. Such considerations refer to donor consent and problems associated to oocyte collection and the issue of destruction of human embryos. Guidelines--ranging from total prohibition to controlled permissiveness--defining what may be permitted in research with pluripotent stem cells have been issued in countries all over the world.

Bioethical aspects will be required related to the scientific and therapeutic relevance and cost of cryopreservation over time, but specially with respect to embryos which may ultimately be used as source of embryonic stem cells, in which case the bioethical conflict may be further aggravated. Also, a regulatory framework will be required to ensure patient accessibility to products and governmental assistance for their regulation and control.

## Competing interests

The author declares that he has no competing interests. The author is Principal Investigator of a preclinical project (not clinical trial) on gene and cell therapy for treatment of haemophilia.

## Authors' contributions

AL has conceived the manuscript, and its design. The author has made intellectual contributions and has made the acquisition, analysis and interpretation of literature data, drafting the manuscript and the final revised manuscript.
